# No Evidence of Association between HIV-1 and Malaria in Populations with Low HIV-1 Prevalence

**DOI:** 10.1371/journal.pone.0023458

**Published:** 2011-08-12

**Authors:** Diego F. Cuadros, Adam J. Branscum, Gisela García-Ramos

**Affiliations:** 1 Department of Biology, University of Kentucky, Lexington, Kentucky, United States of America; 2 Department of Public Health, Oregon State University, Corvallis, Oregon, United States of America; Universidade Federal de Minas Gerais, Brazil

## Abstract

**Background:**

The geographic overlap between HIV-1 and malaria has generated much interest in their potential interactions. A variety of studies have evidenced a complex HIV-malaria interaction within individuals and populations that may have dramatic effects, but the causes and implications of this co-infection at the population level are still unclear. In a previous publication, we showed that the prevalence of malaria caused by the parasite *Plasmodium falciparum* is associated with HIV infection in eastern sub-Saharan Africa. To complement our knowledge of the HIV-malaria co-infection, the objective of this work was to assess the relationship between malaria and HIV prevalence in the western region of sub-Saharan Africa.

**Methodology/Principal Findings:**

Population-based cross-sectional data were obtained from the HIV/AIDS Demographic and Health Surveys conducted in Burkina Faso, Ghana, Guinea, Mali, Liberia and Cameroon, and the malaria atlas project. Using generalized linear mixed models, we assessed the relationship between HIV-1 and *Plasmodium falciparum* parasite rate (*Pf*PR) adjusting for important socio-economic and biological cofactors. We found no evidence that individuals living in areas with stable malaria transmission (*Pf*PR>0.46) have higher odds of being HIV-positive than individuals who live in areas with *Pf*PR≤0.46 in western sub-Saharan Africa (estimated odds ratio 1.14, 95% confidence interval 0.86–1.50). In contrast, the results suggested that *Pf*PR was associated with being infected with HIV in Cameroon (estimated odds ratio 1.56, 95% confidence interval 1.23–2.00).

**Conclusion/Significance:**

Contrary to our previous research on eastern sub-Saharan Africa, this study did not identify an association between *Pf*PR and infection with HIV in western sub-Saharan Africa, which suggests that malaria might not play an important role in the spread of HIV in populations where the HIV prevalence is low. Our work highlights the importance of understanding the epidemiologic effect of co-infection and the relevant factors involved in this relationship for the implementation of effective control strategies.

## Introduction

Malaria has been proposed as an important facilitator for the spread of HIV-1 in areas where both infections geographically overlap [1], [2], [3], [4], [5]. This parasitic infection is one of the most prevalent infectious diseases worldwide. Of the estimated 300 million cases per year, 90% occur in sub-Saharan Africa [6]. Most of the African continent is considered to have stable malaria transmission, with prevalence that exceed the threshold (40%) above which theory predicts it is unlikely that malaria transmission can be interrupted with insecticide-treated bed nets alone [7]. This region covers 8.5 million km^2^ of the continent, with 354.28 million people at risk. Areas with malaria prevalence between 5% and 40%, the range in which theory predicts that malaria transmission could be reduced with insecticide-treated bed nets, cover 5.6 million km^2^ of the continent, with 196.83 million people at risk.

Sub-Saharan Africa has also experienced a severe HIV-1 epidemic, with an estimated 25 million infections over the past two decades [8]. The epidemic, however, is not homogeneous, and its spread has been more intense in the southern and eastern part of the continent, with the western region experiencing a slower growing and less severe epidemic. The causes of this difference are not well understood [9]. In the western part of the continent, the epidemic seems to be more concentrated in the high-risk (core) groups, such as female sexual workers and individuals with high number of sexual partners, while the epidemic is more widely distributed across the general population in countries located in the east and south part of the continent.

In previous work [10], we showed that the prevalence of malaria caused by the parasite *Plasmodium falciparum* is associated with HIV-1 infection in eastern sub-Saharan Africa. Our results indicated that individuals who lived in areas with high *P. falciparum* parasite rate (*Pf*PR>0.42) were approximately twice as likely to be infected with HIV than individuals who lived in areas with low *P. falciparum* parasite rate (*Pf*PR≤0.10) [men: estimated odds ratio (OR) 2.24, 95% confidence interval (CI) 1.62–3.12; women: estimated OR 2.44, 95% CI 1.85–3.21].

In western sub-Saharan Africa, the pattern of the HIV-1 epidemic is different and less severe, yet the prevalence of malaria is high. To examine the association between malaria and the prevalence of HIV-1 under these conditions, and to measure this association in a region with intermediate HIV-1 prevalence in central Africa, we used the most current and comprehensive database, namely the Demographic and Health Survey (DHS) for HIV-1. We also utilized a new source of data for *P. falciparum* malaria endemicity, the malaria atlas project.

To complement our knowledge of HIV-malaria co-infection, the objective of this work was to assess the relationship between malaria and HIV-1 prevalence in the western and central regions of sub-Saharan Africa. The primary aim was to examine the difference in the likelihood of current infection with HIV between individuals who lived in locations with high transmission intensity of the most common vector that transmits malaria in the region, namely the *P. falciparum* parasite, and individuals who lived in areas with low malaria transmission intensity.

## Materials and Methods

### Ethics statement

Our study did not require an ethics committee approval because it relies entirely on previously published data. Written consent was given by the patients enrolled in the previous studies for their information to be stored in the hospital database and used for research.

### HIV data

This study refers to HIV-1 exclusively. We considered the following countries from the western part of sub-Saharan Africa: Burkina Faso, Ghana, Guinea, Liberia and Mali. These countries have similar HIV prevalence (between 1% and 2%), and the collective geographic region has heterogeneous prevalence of malaria for comparisons [7]. We also included Cameroon in a separate analysis to investigate for an association between HIV and malaria in a country with an intermediate HIV prevalence (∼5%) and with similar malaria transmission intensity to the western region included in the study.

To cover the population residing in households in these countries, we extracted data from DHS for 2003 in Burkina Faso (EDSBF-III) [11] and Ghana (GDHS) [12], for 2004 in Cameroon (EDSC-III) [13], for 2005 in Guinea (EDSG-III) [14] , for 2006 in Mali (EDSM-IV) [15], and for 2007 in Liberia (LDHS) [16]. Methods used in these demographic and health surveys have been described in detail elsewhere [13], [14]. Briefly, the surveys used a two-stage cluster sampling technique. The first stage involved selecting sample points (clusters); 400 clusters in Burkina Faso (300 in urban and 100 in rural areas), 412 in Ghana (240 in urban and 172 in rural areas), 297 in Guinea (209 in urban and 88 in rural areas), 408 in Mali (265 in urban and 143 in rural areas), 297 in Liberia (117 in urban and180 in rural areas), and 466 in Cameroon (224 in urban and 242 in rural areas). The global position system (GPS) was used to identify and record the geographic coordinates of each of the DHS clusters.

The second stage used by each DHS included in this study involved the systematic sampling of households from the selected clusters. Adult men (aged 15–59 years) and women (aged 15–49 years) in the selected households were eligible for the survey. In all DHS surveys included in this study, HIV testing was performed using blood samples. The DHS reported that participation in HIV testing was voluntary, and before collecting the samples, each selected participant was asked to provide informed consent to the testing. Further details of the performed HIV test have been described elsewhere [12], [13]. In summary, HIV testing was conducted in a laboratory by following a standard testing algorithm that uses two HIV enzyme immunosorbent assays based on different antigens. Discordant samples that were positive on the first test were retested with the same enzyme immunosorbent assays. A confirmatory HIV Western blot kit was used for all samples that were still discrepant.

We used the HIV serostatus reported by the DHS for each individual as the dichotomous response outcome and restricted the analysis to only sexually active people. After combining all DHS surveys and excluding missing data, the final sample population for data analysis consisted of 41 064 individuals: 18 296 men and 22 768 women for West sub-Saharan Africa, and 10 088 individuals: 4986 men and 5102 women for Cameroon.

### Malaria Data

A commonly used index of malaria transmission intensity is the *P. falciparum* parasite rate (*Pf*PR), defined as the proportion of the population found to carry asexual blood-stage parasites. The enhancement of HIV replication generated by infection with malaria, which supports higher HIV infectivity in co-infected individuals [17], is produced by the presence of any parasitaemia, and not only during clinical episodes [18]. For that reason, this index of malaria transmission might be a measure for use in the initial assessment of the association between malaria and HIV.

For this study, we used the largest and most contemporary spatial database for the *Pf* PR for children aged 2 to 10 years old [7], [19]. This database contains nearly 9000 distinct community surveys across 78 malaria-endemic countries. Many of these surveys, however, reported crude *Pf*PR without stratifying by age. Thus, in previous research [20], an algorithm was developed to age-standardize *Pf*PR data. The *Pf*PR in children aged 2–10 years is correlated with the entomological inoculation rate (the number of infected bites per person per unit of time) [21], and has provided a basis for the most common categorical measures of malaria transmission [20]. Therefore, the *Pf*PR reported by the malaria atlas project is age-standardized to 2–10 years old [7]. Since the *Pf*PR in children aged 2–10 years is related to the entomological inoculation rate, which is an estimate of the force of infection, we used this measure as an approximation of the prevalence of malaria infections in the adult population studied. This assumption, however, has some implications on the study results, which are discussed in the limitations of the study.

To create a continuous surface of malaria endemicity, previous research [7] produced a model-based geostatistical map using a Bayesian framework to incorporate factors such as the spatial density and location of the data, and the number of people sampled in each survey. Geostatistical algorithms generate a continuous map by interpolating values at unsampled locations using a weighted linear combination of the available (neighboring) sample data. Further details on the malaria data and statistical procedures for generating the map have been described elsewhere [7]. To display and extract the *Pf*PR from each DHS cluster, we used the program ArcGIS version 9.2 [22]. From each geo-referenced cluster, we obtained the *Pf*PR and assigned its value to each individual who belonged to that cluster.

### Socio-economic and biological covariates

Socio-economic and biological covariates of potential importance were investigated for inclusion into the final model. To select the covariates to be included in the final model, we first conducted preliminary unadjusted bivariate analyses from a pool of 16 socio-economic and biological variables: age, place of residence (urban or rural), highest educational level, main floor material of the house, main roof material of the house, main wall material of the house, religion, current marital status, previous AIDS testing, wealth index, number of sexual partners in the last year, age at first intercourse, presence of bed net for sleeping, use of a bed net for sleeping, male circumcision, and reported presence of genital ulceration during the last month. Covariates of a priori importance with *p*<0.2 in the unadjusted analyses were included in the final multivariable model relating HIV and malaria.

Several factors were included as categorical variables. Marital status was comprised of three categories: never married, currently married and formerly married. Wealth index is an ordinal variable that characterizes standard of living as determined by material possessions. The DHS calculated the living standard of a household based on relevant assets such as television and bicycles, materials used for housing construction, and the availability of amenities such as electricity and source of drinking water. The resulting asset scores, constructed using principal component analysis, were then used to define wealth quintiles: poorest, poorer, middle, richer, richest [13], [14]. Religion was subdivided into four categories: Muslim, Catholic, traditional religion, and other religions. Education level was included as a categorical variable with four levels: no education, primary education, secondary education and higher education.

### Statistical analysis

Data analysis used generalized linear mixed models (GLMM), specifically logistic regression models with normally distributed random cluster effects to include covariates and account for correlated data among individuals within the same cluster. We first conducted unadjusted analyses for each covariate. We then generated an adjusted model, where the *Pf*PR was categorized into two transmission intensity levels, namely areas with stable malaria transmission (*Pf*PR>0.46), which corresponds to areas with *Pf*PR higher than the first quartile, and areas with *Pf*PR≤0.46. We analyzed the data from the western region and Cameroon separately. All statistical analyses were conducted using R version 2.11.1 [23].

## Results

### West sub-Saharan Africa

The HIV prevalence in responders included in the study for western sub-Saharan Africa was 1.53%: 1.15% for men and 1.80% for women (Table S1). The mean *Pf*PR in the region was 0.52, with a standard deviation of 0.12. In areas with stable malaria transmission (*Pf*PR>0.46) the HIV prevalence was 1.48%, whereas in areas with *Pf*PR≤0.46 the HIV prevalence was 1.58% (Table S1).

The final model included age, gender, urban or rural residence, education, religion, wealth index, marital status and the presence of genital ulcerations. Male circumcision was not significantly associated with being HIV-positive in men (unadjusted OR 0.63, 95% 0.31–1.26). A similar result has been reported by other studies [24], [25], and might be a consequence of the extremely high prevalence of male circumcision in the area of study. We estimated that 94% of the male individuals from the western region and 95% of the male individuals from Cameroon were circumcised. For that reason, we combined the data for men and women into a single analysis, and included gender to adjust for confounding in the final models.

The results indicated no evidence that *Pf*PR was associated with being HIV-positive in western sub-Saharan Africa, where we observed no statistical difference in the odds of being HIV-positive between individuals who lived in areas with stable malaria transmission (*Pf*PR>0.46) and individuals who lived in areas with *Pf*PR≤0.46 (estimated OR 1.14, 95% CI 0.86–1.50). Other categorizations of *Pf*PR, such as grouping by quartiles, gave the same qualitative conclusions.

Men have almost half the likelihood of being HIV seropositive compared to women (estimated OR 0.63, 95% CI 0.51–0.78), which could be connected to the protective effect of male circumcision for HIV acquisition. As observed in eastern sub-Saharan Africa [10], age was found to be strongly associated with HIV infection. The HIV prevalence peaked at age group 30–34 years, and these individuals had higher odds of being HIV seropositive compared with the age group 15–19 years (estimated OR 4.11, 95% CI 2.60–6.51).

As expected and in agreement with other studies [26], [27], the adjusted model indicated that individuals living in rural areas had lower odds of being HIV-positive compared to those living in urban areas, with an estimated OR of 0.58 (95% CI 0.43–0.78). Unadjusted analysis indicated that reported genital ulcerations during the last 12 months increased the likelihood of current HIV infection (estimated OR 1.65, 95% CI 1.21–2.26). The presence of genital ulcerations, however, was not a significant factor after adjusting for the other variables (Table S2). Following the same pattern observed in the eastern part of the continent, being currently married was not a significant factor compared with individuals that had never been married (estimated OR 1.17, 95% CI 0.85–1.63), whereas individuals formerly married had higher odds of having HIV compared with individuals never married (estimated OR 2.55, 95% CI 1.73–3.77).

Among the socio-economic covariates considered, the lone discrepancy between the current study and results obtained from our previous study of eastern sub-Saharan Africa [10] was the relationship between wealth index and HIV. While in the eastern part of the continent the wealth index was found to be positively and monotonically associated with HIV, where individuals in the richest category had the highest odds of being HIV-positive compared with individuals in the poorest category [10], [26], [27], we observed no significant association between wealth index and current HIV status in western sub-Saharan Africa (Table S2).

### Cameroon

The general HIV prevalence in Cameroon was 5.4%: 4.0% for men and 6.7% for women (Table S1). The mean *Pf*PR in this country was 0.44, slightly lower than the *Pf*PR estimated for the western region. In areas with stable malaria transmission (*Pf*PR>0.46) the HIV prevalence was 6.4%, whereas in areas with *Pf*PR≤0.46 the HIV prevalence was 4.1% (Table S1).

The adjusted analysis indicated that *Pf*PR was associated with being HIV-positive in Cameroon and suggested that individuals who lived in areas with stable malaria transmission had increased odds of being HIV-positive compared to individuals who lived in areas with *Pf*PR≤0.46 (estimated OR 1.56, 95% CI 1.23–2.00). The difference in magnitude of the association between *Pf*PR and current HIV status for Cameroon and west sub-Saharan Africa was also identified in an analysis that included an interaction term between *Pf*PR and region (western sub-Saharan or Cameroon) in an adjusted model. The significant interaction term (*p* = 0.04) indicated that the magnitude of the association between HIV and the *Pf*PR was different among these regions. Unlike the countries from the western region included in the study, higher wealth index was associated with higher odds of being HIV seropositive compared to individuals in the poorest category (Table S3), resembling the relationship between wealth index and HIV observed in East sub-Saharan Africa.

## Discussion

In contrast to the observations from eastern sub-Saharan Africa, the results from this study suggest the absence of evidence for an association between malaria transmission intensity and current infection with HIV in western sub-Saharan Africa. The lower HIV prevalence in the western region compared to the eastern region of the continent might be a contributing factor for this difference. Unlike the eastern region, where the HIV epidemic is present in the general population, the low HIV prevalence observed in western countries might indicate that the HIV epidemic is concentrated in high-risk groups.

Our collective work provides evidence that the epidemiology of co-infection is not a simple additive relationship. The effect of co-infection on the spread of HIV might be influenced by other external factors such as the stage of the epidemic, individual and community behavior, and the HIV prevalence in the population, among others. Mathematical models have suggested that the number of HIV infections attributable directly to co-infection, estimated as the population attributable fraction, depends on the stage of the HIV epidemic and the epidemiology of the parasite involved in the co-infection (Cuadros *et al.*, submitted for publication) [28], [29], [30]. These models indicate that co-infections with sexually transmitted infections prevalent in high-risk groups, such as syphilis, trichomoniasis, gonorrhea and chlamydia, have the highest impact on the spread of HIV when the epidemic is concentrated in these subgroups. On the other hand, infections that are highly prevalent in the general population, such as herpes simplex virus type 2 and malaria, are more important contributors to fueling the spread of HIV infection when the epidemic has invaded the general population.

The results from this study and our previous work are thus consistent with the results obtained from mathematical models and indicate that co-occurrence of HIV and infections present in the general population, like malaria, might not play an important role in the spread of HIV in populations where its prevalence is low. Malaria apparently acts as a facilitator and fuels the spread of HIV when the epidemic is present in the general population but might not be the cause of the invasion of HIV in the general population. This hypothesis is supported by the results obtained from data for Cameroon, where regardless of the similar *Pf*PR and prevalence of male circumcision compared to the studied western region, the higher HIV prevalence in Cameroon was observed together with a significant association between HIV and malaria.

In agreement with other studies [9], [31], we found no difference in the socio-economic and demographic cofactors for HIV infection between the eastern and western regions of sub-Saharan Africa. Circumcision, however, was not significantly associated with HIV infection for men in our study. Circumcision is almost universal in western sub-Saharan Africa [32], where 94% of the male individuals included in our study were circumcised. In contrast, only 62% of the male individuals included in the study for the eastern region were circumcised. This difference has been proposed as a key factor for the difference between both epidemics [31], [32]. Circumcision is a cultural practice, and therefore it is plausible to hypothesize that the current prevalence of circumcision might be similar to the prevalence at the early stage of the HIV epidemic in sub-Saharan Africa. Consequently, the high rate of male circumcision observed in western sub-Saharan Africa could have generated a protective effect that prevented the invasion of the epidemic to the general population.

The results from Cameroon, however, did not support this hypothesis. Despite the extent of male circumcision (95%), the higher HIV prevalence indicates the likely presence of an HIV epidemic in the general population, which in turn might have governed the interaction between HIV and malaria that is suggested by our results. Thus, the factors (biological and behavioral) that trigger the movement of an HIV epidemic from high-risk groups to the general population remain indeterminate. The identification of these factors may give us a better understanding of the geographical differences observed in the HIV epidemic and a better comprehension of the complex co-infection relationship between HIV and other parasites.

The present work as well as our previous study [10] indicate that the presence of a parasite influencing the transmission of HIV, even when the parasite is highly prevalent in the population, does not fully explain the interaction and the outcome of co-infection on the spread of HIV. Other factors such as the HIV prevalence and the distribution of the infection in the population may dictate the role of co-infection in fueling the epidemic.

Our work highlights the importance of identifying factors for the implementation of effective control interventions focused on co-infection. The effect of co-infection might not be the same in different populations, and control strategies will not necessarily have the same impact in each population. Understanding the epidemiological effects of co-infection and the relevant factors involved in this relationship is a prerequisite to developing accurate and effective recommendations for population-level control strategies.

### Limitations of the study

Although our study makes use of the most complete and contemporary databases for both HIV and malaria, it is important to emphasize that our results were based on geographical estimates of *Pf*PR in children aged 2 to 10 years old in locations where DHS surveys were made, and thus we estimated the effect of malaria on the prevalence of HIV based on indirect measures of malaria transmission intensity. Therefore, the results obtained in this study depend on the quality of these secondary data and on the methodology implemented in our previous analyses. Consequently, they should be interpreted with caution.

Furthermore, it is important to highlight that our estimates are based on prevalence data for HIV. Since HIV prevalence is highly influenced by other independent factors, such as the stage and distribution of the epidemic in the population, this epidemiological measure might not be the most appropriate for evaluating the interaction between both infections. We used this measure based on the availability and quality of the data derived from different DHS surveys. Other epidemiological estimates such as HIV incidence, however, would be a more appropriate measure for direct estimation of the association between incident HIV and malaria. Therefore, our collective work represents a preliminary step to elucidate the role of malaria on the HIV epidemic in sub-Saharan Africa, and highlights the necessity of more appropriate data for understanding the malaria-HIV relationship in order to implement effective control interventions.

On the other hand, the *Pf*PR values used in our study were obtained by mathematical algorithms that standardized the malaria transmission intensity in children aged 2–10 years [21]. Whereas this estimation is a widely accepted approximation of the malaria transmission intensity in a specific region [20], it is important to note that most HIV infections occur in the adult population. Repetitive malaria infections during childhood generate partial immunity to the infection and thus decrease the prevalence of clinical malaria in adults, especially in areas where malaria is endemic [33]. Although the *Pf*PR in children aged 2–10 has been widely accepted as a measure of the transmission intensity, this variable is only an approximation to the malariological index. Furthermore, the relationship between this variable and the transmission intensity in the adult population is still not well defined, and most likely the *Pf*PR in adults would be somewhat different. For that reason, it is important to note that other malariological measures, such as the entomological inoculation rate or the prevalence of malaria in the adult population, would be alternative ways to investigate for the association between the co-infection studied here. These alternative data, however, are scarce, whereas the database for malaria used here allowed us to obtain the proxy data necessary for performing the analysis in this study. Moreover, we expect that the geographic distribution of the intensity of malaria transmission remains the same for all populations (children and adults), in which case the geographic variability of the *Pf*PR used here represents a useful approximation for the comparisons made in this study.

Additionally, the relationship between malaria and HIV is bilateral, where HIV infection might alter the natural history of malaria (and vice versa), especially in areas with high HIV prevalence and unstable malaria transmission [34], [35], [36]. In our study, however, we focused on the association between an ecological proxy of malaria infection and current HIV infection rather than the potential effect of HIV on malaria. Since the region studied is characterized by an endemic malaria epidemic [19] and low prevalence of HIV, we expect that this assumption would not drastically affect the outcome of our study.

## Supporting Information

Table S1 **HIV prevalence by socio-economic and biological characteristics in western sub-Saharan Africa and Cameroon.**
(PDF)

Table S2 **Unadjusted and adjusted results from western sub-Saharan Africa: HIV serostatus according to selected socio-economic and biological characteristics.**
(PDF)

Table S3 **Unadjusted and adjusted results from Cameroon: HIV serostatus according to selected socio-economic and biological characteristics.**
(PDF)

## References

[1] Abu-Raddad LJ, Patnaik P, Kublin JG (2006). Dual infection with HIV and malaria fuels the spread of both diseases in sub-Saharan Africa.. Science.

[2] Herrero MD, Rivas P, Rallon N, Ramirez-Olivencia G, Puente S (2007). HIV and malaria.. AIDS Rev.

[3] Idemyor V (2007). Human immunodeficiency virus (HIV) and malaria interaction in sub-Saharan Africa: The collision of two titans.. HIV Clin Trials.

[4] Kublin JG, Steketee RW (2006). HIV infection and malaria - Understanding the interactions.. J Infec Dis.

[5] Renia L, Potter SM (2006). Co-infection of malaria with HIV: an immunological perspective.. Parasite Immunol.

[6] Snow RW, Guerra CA, Noor AM, Myint HY, Hay SI (2005). The global distribution of clinical episodes of Plasmodium falciparum malaria.. Nature.

[7] Hay SI, Guerra CA, Gething PW, Patil AP, Tatem AJ (2009). A world malaria map: Plasmodium falciparum endemicity in 2007.. Plos Med.

[8] WHO Joint United Nations Programme on HIV/AIDS (UNAIDS)/World Health Organization “AIDS epidemic update 2005”.. http://www.unaids.org/epi/2005/doc/report_pdf.asp.

[9] Buve A, Carael M, Hayes RJ, Auvert B, Ferry B (2001). The multicentre study on factors determining the differential spread of HIV in four African cities: summary and conclusions.. AIDS.

[10] Cuadros DF, Branscum AJ, Crowley PH (2011). HIV–malaria co-infection: effects of malaria on the prevalence of HIV in East sub-Saharan Africa.. Int J Epidemiol.

[11] Institut National de la Statistique et de la Démographie MdlÉedD O, Burkina Faso (2004). Enquête Démographique et de Santé.

[12] Ghana Statistical Service NMIfMR ORC Macro (2004). Ghana Demographic and Health Survey 2003.

[13] National Statistical Office aOM (2005). Cameroon Demographic and Health Survey 2004.

[14] National Statistical Office CG and ORC Macro (2006). Guinea Demographic and Health Survey 2005.

[15] Cellule de Planification et de Statistique Ministère de la Santé DNdlSedlI Ministère de l'Économie, de l'Industrie et du Commerce Bamako, Mali, Macro International Inc (2007).

[16] Liberia Institute of Statistics and Geo-information Services LISGIS ORC Macro (2007). Liberia Demographic and Health Survey 2007.

[17] Quinn TC, Wawer MJ, Sewankambo N, Serwadda D, Li CJ (2000). Viral load and heterosexual transmission of human immunodeficiency virus type 1.. New Engl J Med.

[18] Kublin JG, Patnaik P, Jere CS, Miller WC, Hoffman IF (2005). Effect of Plasmodium falciparum malaria on concentration of HIV-1-RNA in the blood of adults in rural Malawi: a prospective cohort study.. Lancet.

[19] Hay SI, Snow RW (2006). The malaria atlas project: Developing global maps of malaria risk.. Plos Med.

[20] Smith DL, Guerra CA, Snow RW, Hay SI (2007). Standardizing estimates of the Plasmodium falciparum parasite rate.. Malaria J.

[21] Hay SI, Smith DL, Snow RW (2008). Measuring malaria endemicity from intense to interrupted transmission.. Lancet Infect Dis.

[22] ESRI (2004). ArcGIS 9.x.

[23] R Development Core Team (2010). R: A language and environment for statistical computing. 2.11.1 ed.

[24] De Walque D (2006). Who gets AIDS and how?.

[25] Mishra V, Assche SRV, Greener R, Vaessen M, Hong R (2007). HIV infection does not disproportionately affect the poorer in sub-Saharan Africa.. AIDS.

[26] Msisha WM, Kapiga SH, Earls F, Subramanian S (2008). Socioeconomic status and HIV seroprevalence in Tanzania: a counterintuitive relationship.. Int J Epidemiol.

[27] Johnson K, Way A (2006). Risk factors for HIV infection in a national adult population: Evidence from the 2003 Kenya demographic and health survey.. JAIDS.

[28] Abu-Raddad LJ, Magaret AS, Celum C, Wald A, Longini IM (2008). Genital herpes has played a more important role than any other sexually transmitted infection in driving HIV prevalence in Africa.. PLoS ONE.

[29] Freeman EE, Orroth KK, White RG, Glynn JR, Bakker R (2007). Proportion of new HIV infections attributable to herpes simplex 2 increases over time: simulations of the changing role of sexually transmitted infections in sub-Saharan African HIV epidemics.. Sex Transm Infect.

[30] Orroth KK, Freeman EE, Bakker R, Buve A, Glynn JR (2007). Understanding the differences between contrasting HIV epidemics in east and west Africa: results from a simulation model of the Four Cities Study.. Sex Transm Infect.

[31] Auvert B, Buve A, Ferry B, Carael M, Morison L (2001). Ecological and individual level analysis of risk factors for HIV infection in four urban populations in sub-Saharan Africa with different levels of HIV infection.. AIDS.

[32] Auvert B, Buve A, Lagarde E, Kahindo M, Chege J (2001). Male circumcision and HIV infection in four cities in sub-Saharan Africa.. AIDS.

[33] Kamya MR, Gasasira AF, Yeka A, Bakyaita N, Nsobya SL (2006). Effect of HIV-1 infection on antimalarial treatment outcomes in Uganda: a population-based study.. J Infect Dis.

[34] Korenromp EL, Williams BG, de Vlas SJ, Gouws E, Gilks CF (2005). Malaria attributable to the HIV-1 epidemic, sub-Saharan Africa.. Emerg Infect Dis.

[35] Grimwade K, French N, Mbatha DD, Zungu DD, Dedicoat M (2004). HIV infection as a cofactor for severe falciparum malaria in adults living in a region of unstable malaria transmission in South Africa.. AIDS.

[36] Whitworth J, Morgen D, Quigley M, Smith A, Mayanja S (2000). Effect of HIV-1 and increasing immunosuppression on malaria parasitaemia and clinical episodes in adults in rural Uganda: a cohort study.. Lancet.

