# Supplementary material for: No Evidence of Association between HIV-1 and Malaria in Populations with Low HIV-1 Prevalence
Source: PLoS One. 2011 Aug 12;6(8):e23458. doi: 10.1371/journal.pone.0023458 (PMC3155564; doi:10.1371/journal.pone.0023458)
Supplement: Table S1 — HIV prevalence by socio-economic and biological characteristics in western sub-Saharan Africa and Cameroon. (PDF) [file pone.0023458.s001.pdf]

Table S1. HIV prevalence by socio-economic and biological characteristics in western sub-Saharan Africa and Cameroon.

Characteristics	Western Africa<br>N (% HIV-positive)	Cameroon<br>N (% HIV-positive)
**Malaria**		
*Pf*PR ≤ 0.46	10 207 (1.58)	4871 (4.1)
*Pf*PR > 0.46	30 857 (1.48)	5217 (6.4)
**Gender**		
Female	22 768 (1.80)	5102 (6.7)
Male	18 296 (1.15)	4986 (4.0)
**Age**		
15-19	8690 (0.52)	2443 (1.4)
20-24	6844 (1.18)	1940 (5.0)
25-29	6006 (2.00)	1582 (8.2)
30-34	5247 (2.19)	1245 (8.5)
35-39	5009 (2.15)	966 (8.3)
40-49	7614 (1.60)	1476 (5.6)
50-59	1654 (1.75)	436 (2.0)
**Place of residence**		
Urban	13 589 (2.25)	4887 (6.6)
Rural	27 475 (1.16)	5201 (4.3)
**Marital status**		
Never married	11 575 (0.83)	3160 (2.7)
Currently married	27 349 (1.60)	6013 (5.8)
Formerly married	2140 (4.06)	915 (12.0)
**Religion**		
Muslim	19 884 (1.31)	1628 (4.70)
Christian	12 755 (1.65)	4051 (5.60)
Traditional religion	388 (0.25)	271 (1.85)
Other	8037 (1.87)	4138 (5.58)

**Wealth index**		
Poorest	8469 (1.02)	1569 (2.9)
Poorer	8235 (1.25)	1819 (3.2)
Middle	8219 (1.27)	2264 (6.0)
Richer	7937 (1.88)	2189 (7.0)
Richest	8204 (2.17)	2247 (6.7)
**Highest educational level**		
None	21 502 (1.30)	1504 (3.6)
Primary school	8460 (1.45)	4007 (5.7)
Secondary	10 268 (1.94)	4217 (5.9)
Higher	834 (1.99)	360 (4.4)
**Genital ulceration**		
No genital ulceration last 12 months	40 644 (1.42)	9843 (5.5)
Genital ulceration last 12 months	420 (2.22)	245 (9.7)
**Male circumcision**		
No circumcised	1018 (1.79)	253 (1.59)
Circumcised	17 278 (1.31)	4733 (4.18)
