# Supplementary material for: No Evidence of Association between HIV-1 and Malaria in Populations with Low HIV-1 Prevalence
Source: PLoS One. 2011 Aug 12;6(8):e23458. doi: 10.1371/journal.pone.0023458 (PMC3155564; doi:10.1371/journal.pone.0023458)
Supplement: Table S2 — Unadjusted and adjusted results from western sub-Saharan Africa: HIV serostatus according to selected socio-economic and biological characteristics. (PDF) [file pone.0023458.s002.pdf]

Table S2. Unadjusted and adjusted results from western sub-Saharan Africa: HIV serostatus according to selected socio-economic and biological characteristics.

	OR (95% CI)	
**Indicator**	Unadjusted	Adjusted
**Malaria**		
*Pf*PR ≤ 0.46	1.00	1.00
*Pf*PR > 0.46	0.95 (0.74-1.21)	1.14 (0.86-1.50)
**Gender**		
Female	1.00	1.00
Male	0.64 (0.53-0.77)	0.63 (0.51-0.78)
**Age**		
15-19	1.00	1.00
20-24	2.39 (1.58-3.62)	2.12 (1.38-3.25)
25-29	4.18 (2.82-6.17)	3.66 (2.35-5.68)
30-34	4.68 (3.16-6.94)	4.11 (2.60-6.51)
35-39	4.62 (3.10-6.87)	4.18 (2.62-6.67)
40-49	3.46 (2.34-5.12)	3.14 (1.97-4.99)
50-59	3.91 (2.28-6.72)	5.30 (2.84-9.88)
**Place of residence**		
Urban	1.00	1.00
Rural	0.48 (0.39-0.60)	0.58 (0.43-0.78)
**Marital status**		
Never married	1.00	1.00
Currently married	2.19 (1.70-2.82)	1.17 (0.85-1.63)
Formerly married	5.45 (3.90-7.62)	2.55 (1.73-3.77)
**Religion**		
Muslim	1.00	1.00
Christian	1.28 (1.01-1.62)	1.11 (0.86-1.46)
Traditional religion	0.24 (0.02-2.45)	0.28 (0.02-2.87)
Other	1.39 (1.07-1.80)	1.24 (0.94-1.63)

**Wealth index**		
Poorest	1.00	1.00
Poorer	1.20 (0.85-1.69)	1.15 (0.82-1.62)
Middle	1.21 (0.86-1.70)	1.10 (0.78-1.56)
Richer	1.81 (1.31-2.51)	1.40 (0.97-2.01)
Richest	2.01 (1.45-2.77)	1.38 (0.92-2.08)
**Highest educational level**		
None	1.00	1.00
Primary school	1.03 (0.80-1.32)	1.21 (0.93-1.57)
Secondary	1.33 (1.07-1.66)	1.38 (1.06-1.79)
Higher	1.31 (0.74-2.29)	1.01 (0.56-1.82)
**Genital ulceration**		
No genital ulceration last 12 months	1.00	1.00
Genital ulceration last 12 months	1.65 (1.21-2.26)	1.27 (0.93-1.76)
