# Supplementary material for: No Evidence of Association between HIV-1 and Malaria in Populations with Low HIV-1 Prevalence
Source: PLoS One. 2011 Aug 12;6(8):e23458. doi: 10.1371/journal.pone.0023458 (PMC3155564; doi:10.1371/journal.pone.0023458)
Supplement: Table S3 — Unadjusted and adjusted results from Cameroon: HIV serostatus according to selected socio-economic and biological characteristics. (PDF) [file pone.0023458.s003.pdf]

Table S3. Unadjusted and adjusted results from Cameroon: HIV serostatus according to selected socio-economic and biological characteristics.

	OR (95% CI)	
**Indicator**	Unadjusted	Adjusted
**Malaria**		
*Pf*PR ≤ 0.46	1.00	1.00
*Pf*PR > 0.46	1.64 (1.29-2.07)	1.56 (1.23-2.00)
**Gender**		
Female	1.00	1.00
Male	0.55 (0.46-0.67)	0.58 (0.47-0.71)
**Age**		
15-19	1.00	1.00
20-24	4.05 (2.67-6.13)	3.59 (2.33-5.52)
25-29	6.65 (4.42-9.98)	5.86 (3.76-9.14)
30-34	7.07 (4.66-10.71)	6.07 (3.82-9.63)
35-39	6.45 (4.18-9.95)	5.65 (3.48-9.15)
40-49	4.58 (2.99-7.02)	3.98 (2.46-6.43)
50-59	1.36 (0.60-3.10)	1.72 (0.73-4.09)
**Place of residence**		
Urban	1.00	1.00
Rural	0.63 (0.50-0.78)	0.76 (0.57-1.01)
**Marital status**		
Never married	1.00	1.00
Currently married	2.41 (1.86-3.13)	1.17 (0.86-1.60)
Formerly married	5.39 (3.95-7.37)	2.66 (1.87-3.79)
**Religion**		
Muslim	1.00	1.00
Christian	1.17 (0.86-1.59)	0.94 (0.67-1.30)
Traditional religion	0.45 (0.17-1.20)	0.51 (0.19-1.37)
Other	1.14 (0.84-1.55)	0.92 (0.66-1.27)

**Wealth index**		
Poorest	1.00	1.00
Poorer	0.99 (0.64-1.51)	0.88 (0.57-1.36)
Middle	1.94 (1.33-2.83)	1.66 (1.12-2.47)
Richer	2.26 (1.55-3.29)	1.90 (1.24-2.93)
Richest	2.09 (1.43-3.06)	1.70 (1.06-2.93)
**Highest educational level**		
None	1.00	1.00
Primary school	1.44 (1.03-2.02)	1.33 (0.91-1.94)
Secondary	1.45 (1.03-2.04)	1.24 (0.82-1.86)
Higher	1.05 (0.57-1.96)	0.7 (0.37-1.43)
**Genital ulceration**		
No genital ulceration last 12 months	1.00	1.00
Genital ulceration last 12 months	1.81 (1.14-2.88)	1.39 (0.87-2.22)
